# Mutant KRAS Drives Immune Evasion by Sensitizing Cytotoxic T‐Cells to Activation‐Induced Cell Death in Colorectal Cancer

**DOI:** 10.1002/advs.202203757

**Published:** 2023-01-04

**Authors:** Huashan Liu, Zhenxing Liang, Sijing Cheng, Liang Huang, Wenxin Li, Chi Zhou, Xiaobin Zheng, Shujuan Li, Ziwei Zeng, Liang Kang

**Affiliations:** ^1^ Department of Colorectal Surgery and Guangdong Provincial Key Laboratory of Colorectal and Pelvic Floor Diseases The Sixth Affiliated Hospital Sun Yat‐sen University Guangzhou Guangdong 510655 P. R. China; ^2^ School of Medicine Sun Yat‐sen University Shenzhen Guangdong 518107 P. R. China; ^3^ State Key Laboratory of Oncology in South China Collaborative Innovation Center for Cancer Medicine Sun Yat‐sen University Cancer Center Guangzhou 510060 P. R. China; ^4^ Department of Colorectal Surgery Sun Yat‐sen University Cancer Center Guangzhou 510060 P. R. China; ^5^ Department of Pharmacy The Third Affiliated Hospital of Zhengzhou University Zhengzhou Henan 450052 P. R. China; ^6^ University Clinic Mannheim Medical Faculty Mannheim Heidelberg University 68167 Mannheim Germany

**Keywords:** activation‐induced cell death, immune evasion, mutant KRAS

## Abstract

The roles of oncogenic KRAS in tumor immune evasion remain poorly understood. Here, mutant KRAS is identified as a key driver of tumor immune evasion in colorectal cancer (CRC). In human CRC specimens, a significant reduction in cytotoxic CD8^+^ T‐cell tumor infiltration is found in patients with mutant versus wild type KRAS. This phenomenon is confirmed by preclinical models of CRC, and further study showed KRAS mutant tumors exhibited poor response to anti‐PD‐1 and adoptive T‐cell therapies. Mechanistic analysis revealed lactic acid derived from mutant KRAS‐expressing tumor cells sensitized tumor‐specific cytotoxic CD8^+^ T‐cells to activation‐induced cell death via NF‐*κ*B inactivation; this may underlie the inverse association between intratumoral cytotoxic CD8^+^ T‐cells and KRAS mutation. Importantly, KRAS mutated tumor resistance to immunotherapies can be overcome by inhibiting KRAS or blocking lactic acid production. Together, this work suggests the KRAS‐mediated immune program is an exploitable therapeutic approach for the treatment of patients with KRAS mutant CRC.

## Graphical abstract

KRAS mutation in colorectal cancer drives immune evasion by sensitizing cytotoxic T‐cells to activation‐induced cell death through lactic acid‐mediated NF‐*κ*B inactivation, and thus impairs immunotherapeutic efficacy. KRAS inhibition or blockade of lactic acid production represents an exploitable approach to improve cancer immunotherapies.

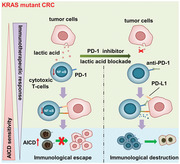

## 1 Introduction

Mutant KRAS represents one of the most frequently tumor driver mutations, and its involvement in multistep tumorigenesis and tumor progression is well recognized.^[^ ^1^ ^]^ The mutated versions of KRAS occur in ≈35–50% of colorectal cancer (CRC) cases, and they typically correlate with disease aggressiveness and poor patient prognosis.^[^ ^2^, ^3^ ^]^ Oncogenic KRAS leads to constitutive oncoprotein activation by interrupting GTPase‐activating protein‐mediated GTP hydrolysis, thereby disrupting the equilibrium between inactive GDP‐bound and active GTP‐bound states.^[^ ^4^, ^5^, ^6^ ^]^ Consequently, mutationally activated KRAS causes aberrant signaling which plays critical roles in tumor pathogenesis and in governing clinical resistance to certain standard‐of‐care first‐line treatments.^[^ ^7^, ^8^, ^9^ ^]^

The prevailing idea regarding KRAS biological mechanisms is that oncogenic KRAS acts in a cell‐intrinsic fashion to sustain its protumoral activities. However, recent studies have indicated the significance of mutant KRAS in engaging with the tumor microenvironment.^[^ ^10^, ^11^, ^12^ ^]^ The cell‐extrinsic manner highlights that mutated KRAS is emerging as a player in engineering a permissive microenvironment for tumor progression. As a major component and pivotal orchestrator of the tumor microenvironment, intratumoral cytotoxic CD8^+^ T‐cells have been shown to correlate with favorable clinical outcomes in CRC patients.^[^ ^13^, ^14^ ^]^ These cells have the potential to recognize and destroy cancer cells during tumor immunosurveillance, and to determine cancer immunotherapeutic efficacy.^[^ ^15^, ^16^ ^]^ However, the role of oncogenic KRAS in tumor‐infiltrating cytotoxic CD8^+^ T‐cell fate decisions has not been fully deciphered. Here, we sought to elucidate the involvement of mutant KRAS in cytotoxic CD8^+^ T‐cell fate decisions and to uncover its functional effects on tumor immune escape.

## 2 Results

### 2.1 Clinical Correlation between Mutant KRAS and Intratumoral Cytotoxic CD8^+^ T‐Cells

**Figure 1:**
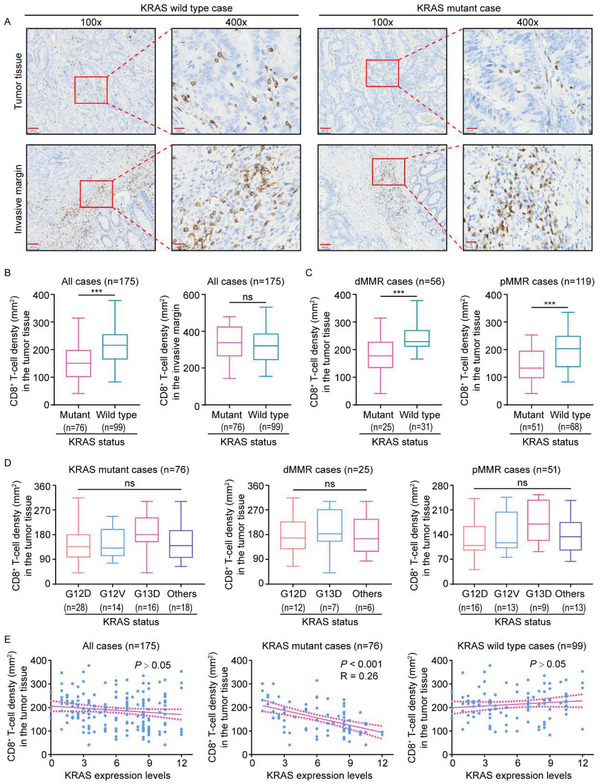
Clinical correlation between mutant KRAS and intratumoral cytotoxic CD8^+^ T‐cells. A) CD8 immunostaining within tumor tissue and invasive margin in a representative human CRC case carrying mutant KRAS compared to a KRAS wild‐type case. B) Quantification of CD8^+^ T‐cells within tumor tissues and invasive margin in KRAS mutant (n = 76) and KRAS wild‐type cases (n = 99). C) Statistical analysis of CD8^+^ T‐cells within tumor tissues in dMMR (n = 56) and pMMR cases (n = 119). D) Statistics of CD8^+^ T‐cells within the tumor tissues in 76 CRC patients of KRAS mutations (Left panel, Others include KRAS G12C (n = 4), G12R (n = 2), G12S (n = 6), G13C (n = 1), K117N (n = 1), A146T (n = 2), and Q61H (n = 2) mutations), and in 25 CRC patients of dMMR (Middle panel, Others include KRAS G12V (n = 1), K117N (n = 1), A146T (n = 2), and Q61H (n = 2) mutations), and in 51 CRC patients of pMMR (Right panel, Others include KRAS G12C (n = 4), G12R (n = 2), G12S (n = 6), and G13C (n = 1) mutations). E) Pearson's correlation analysis between KRAS expression levels and CD8^+^ T‐cell density in CRC tissues. Scare bars, 100 µm (A), ***P ≤ 0.001, and ns indicates *P* > 0.05, by two‐tailed Student's *t*‐test (B,C), one‐way ANOVA (D), or Person's correlation analysis (E).

To specifically probe the potential link between KRAS status and cytotoxic T‐cell tumor infiltrate, we analyzed CD8^+^ T‐cell density in 76 KRAS mutant CRC tissues and 99 KRAS wild‐type samples. Results showed that in addition to hyperactive RAS signaling (Figure S1, Supporting Information), KRAS mutant samples versus KRAS wild‐type counterparts had a significant decrease in CD8^+^ T‐cell density within tumor tissue, but no discernible difference within the invasive margin (**Figure** **1**A,B). These findings suggest an inverse association between mutant KRAS and cytotoxic CD8^+^ T‐cell tumor infiltrate. Cohort stratification into dMMR and pMMR subgroups revealed tumor‐infiltrating cytotoxic CD8^+^ T‐cell reduction could still be seen in KRAS mutant CRC (Figure 1C).

We next investigated whether cytotoxic CD8^+^ T‐cell tumor infiltrates correlated with a specific KRAS mutation type. Results indicated cytotoxic CD8^+^ T‐cell tumor infiltrate was unlikely to correlate with KRAS mutation type by demonstrating comparable tumor‐infiltrating cytotoxic CD8^+^ T‐cell density in CRC tissues with different types of KRAS mutations (Figure 1D). Subsequently, we set out to investigate whether tumor‐infiltrating cytotoxic CD8^+^ T‐cells were linked to KRAS expression levels. Correlation analysis revealed that KRAS expression was not significantly related to CD8^+^ T‐cell density in all cases (Figure 1E). Further analyses stratifying patients according to KRAS status indicated that CD8^+^ T‐cell density was inversely correlated with KRAS expression levels in KRAS mutant, but not wild type, CRC cases (Figure 1E). Hence, we conclude intratumoral cytotoxic CD8^+^ T‐cell reduction is likely dictated by mutant, but not wild type, KRAS.

### 2.2 Mutant KRAS Drives Intratumoral Cytotoxic CD8^+^ T‐Cell Reduction

**Figure 2:**
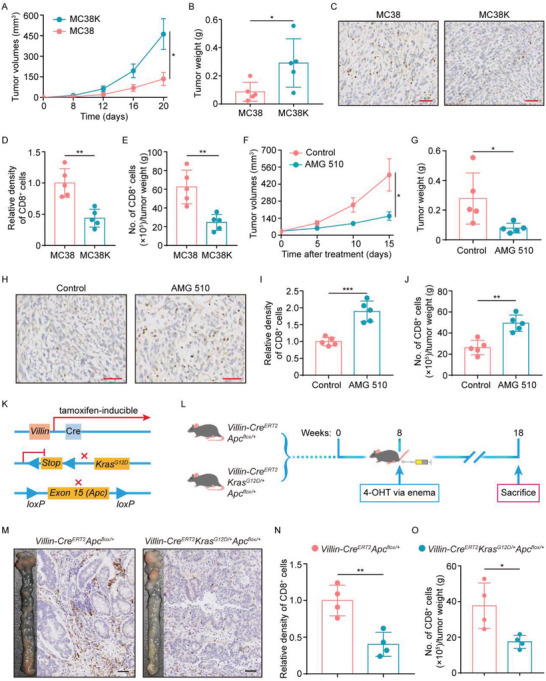
CD8^+^ T‐cell reduction in KRAS mutant tumors. A–E) MC38 and MC38K cells were subcutaneously injected into C57BL/6J mice (n = 5 mice per group). A) Tumor growth curves for the indicated tumors. B) Tumor weights were measured on day 20. C) Representative IHC staining, D) relative densities, and E) flow cytometry analysis of CD8^+^ cells in MC38 versus MC38K tumors. F–J) MC38K cells were subcutaneously injected into C57BL/6J mice. When tumors were palpable, mice were treated with Control or 30 mg kg^−1^ AMG 510 (n = 5 mice per group). F) Tumor growth curves during the course of each indicated treatment. G) Tumor weights measured after 15 days of AMG 510 treatment. H) Representative IHC staining, I) relative densities, and J) flow cytometry analysis of CD8^+^ cells in tumors with the indicated treatments. K,L) Genetic strategy: conditional alleles of Apc, and Kras^G12D^ were crossed with the tamoxifen‐inducible Villin‐Cre^ERT2^. Mice were administrated with 1 mg mL^−1^ 4‐OHT tamoxifen into the adult colon via enema at week 8, and were euthanized at week 18 (n = 4 mice per group). M) Gross inspection of colonic tumorigenesis and representative IHC staining of CD8^+^ cells in Villin‐Cre^ERT2^Apc^flox/+^ versus Villin‐Cre^ERT2^Kras^G12D/+^Apc^flox/+^ mice. N) Relative densities of CD8^+^ cells in tumors harvested from the indicated mice. O) The indicated tumors were analyzed by flow cytometry of CD8^+^ cells. Scare bars, 50 µm (C,H,M). *P ≤ 0.05, **P ≤ 0.01, and ***P ≤ 0.001, by two‐tailed Student's *t*‐test (A,B,D–G,I,J,N,O).

The observed correlation between cytotoxic CD8^+^ T‐cell tumor infiltrate and KRAS mutation in clinical samples does not necessarily indicate mutant KRAS was a causative factor in reducing tumor‐infiltrating cytotoxic CD8^+^ T‐cells. To clarify whether tumor‐infiltrating cytotoxic CD8^+^ T‐cell reduction in KRAS mutant CRC tissue was a consequence of KRAS mutation, the wild‐type Kras‐expressing MC38 cell line was stably transfected with cDNA encoding the Kras^G12C^ to generate the cells expressing Kras^G12C^ (designated as MC38K), while MC38 cells with transfection of the corresponding empty vectors were used as controls (MC38 hereafter). As expected, stable transfection of Kras^G12C^ led to increased Kras expression and hyperactive Ras signaling (Figure S2A,B, Supporting Information), and subcutaneous C57BL/6J mice xenografts showed a significant increase in MC38K tumor growth as opposed to MC38 tumors (**Figure** **2**A,B). In line with clinical sample experiments, cytotoxic CD8^+^ T‐cell tumor infiltrate was significantly decreased in MC38K versus MC38 tumors (Figure 2C–E). More importantly, treatment with KRAS^G12C^ inhibitor AMG 510 in MC38K tumors effectively inhibited tumor growth (Figure 2F,G), and reverted CD8^+^ T‐cell tumor infiltration (Figure 2H–J). In addition, MC38 tumors had no response to AMG 510 (Figure S2C,D, Supporting Information). Together, these results suggest a causal link between mutant KRAS and intratumoral cytotoxic CD8^+^ T‐cell reduction.

To confirm the role of mutant KRAS in driving intratumoral cytotoxic CD8^+^ T‐cell reduction, we established a genetically engineered CRC mouse model (Figure 2K). In this model, conditional Apc alleles with or without LSL‐Kras^G12D^ (Kras^mut^) were crossed with the tamoxifen‐inducible Villin‐Cre^ERT2^, and then 4‐hydroxytamoxifen (4‐OHT) was introduced into the adult colon via enema (Figure 2L). As anticipated, 4‐OHT had the ability to elicit hyperactive Ras signaling (Figure S2E, Supporting Information) and produce colonic tumors in mice harboring a conditional Apc allele (Figure 2M), and particularly, combining Apc and Kras^mut^ markedly increased tumor load (Figure 2M). Notably, cytotoxic CD8^+^ T‐cell density in tumors from mice harboring conditional Apc and Kras^mut^ alleles was significantly decreased compared to that in mice with only conditional Apc alleles (Figure 2M–O). Collectively, these data support the view that mutated KRAS is the causative factor driving intratumoral cytotoxic CD8^+^ T‐cell decrease.

### 2.3 Mutant KRAS Impairs Anti‐PD‐1 and Adoptive T‐Cell Efficacy

**Figure 3:**
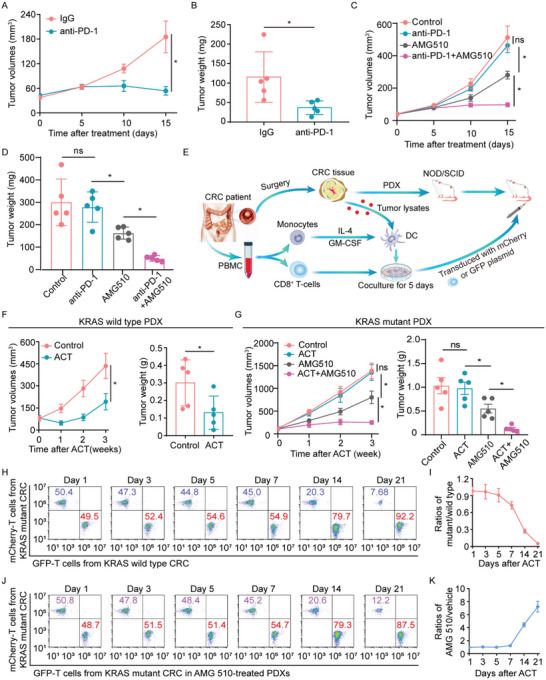
Effects of mutant KRAS on anti‐PD‐1 and ACT efficacy. A,B) MC38 cells were subcutaneously injected into C57BL/6J mice. After palpable tumor formation, mice were treated with IgG or anti‐PD‐1 (n = 5 mice per group). A) Tumor growth curves during the course of each indicated treatment. B) Tumor weights were measured after 15 days of the indicated treatment. C,D) MC38K cells were subcutaneously injected into C57BL/6J mice. When tumors were palpable, mice were treated with anti‐PD‐1 in combination with AMG 510 (n = 5 mice per group). C) Tumor growth curves and D) weights of tumors after the indicated treatment. E) Scheme of ACT by infusing tumor‐reactive T‐cells into NOD.SCID mice transplanted with autologous CRC PDXs. F) Tumor growth curves and weights of KRAS wild‐type PDXs treated with ACT (n = 5 mice per group). G) Tumor growth curves and weights of KRAS mutant PDX tumors treated with ACT in combination with AMG 510 (n = 5 mice per group). H,I) Tumor‐reactive T‐cells from CRC patients with wild type and mutant KRAS were transduced with GFP and mCherry in vitro, respectively. When the PDXs were palpable, GFP‐ or mCherry‐transduced tumor‐reactive T‐cells were transferred into autologous PDXs (n = 3 mice per group). At 1, 3, 5, 7, 14, and 21 days after transfer, KRAS wild‐type PDXs were mixed with an equal weight of KRAS mutant PDXs, and then transferred cells were retrieved from the pooled tissues. Flow cytometry was used to determine the proportion of GFP‐ or mCherry‐transduced T‐cells in CD8^+^ cells. J,K) GFP‐ or mCherry‐transduced tumor‐reactive T‐cells were transferred into KRAS mutant PDXs with or without AMG 510 treatment (n = 3 mice per group). At the indicated time points, KRAS mutant PDXs were mixed with an equal weight of AMG 510‐treated PDXs and then transferred cells were recovered from the pooled tissues. Flow cytometry was used to determine the proportion of GFP‐ or mCherry‐transduced T‐cells in purified total CD8^+^ cells. *P ≤ 0.05, and ns indicates *P* > 0.05, by two‐tailed Student's *t*‐test (A,B,F) or one‐way ANOVA (C,D,G).

According to the above findings, we reasoned the poor response to immunotherapies in CRC may be in part due to the presence of mutant KRAS causing a reduction of tumor‐infiltrating cytotoxic CD8^+^ T‐cells. Hence, we sought to gain insights into the functional significance of KRAS mutation in cancer immunotherapies, including anti‐PD‐1 and adoptive T cell therapies. To this end, MC38 and MC38K cell lines were utilized to establish a subcutaneous xenograft tumor model. MC38 cells demonstrated a strong response to anti‐PD‐1 treatment (**Figure** **3**A,B; Figure S3, Supporting Information). In MC38K tumor‐bearing mice, significant antitumoral effects of anti‐PD‐1 therapy were not found (Figure 3C,D; Figure S3, Supporting Information), but the anti‐PD‐1 responses in MC38K tumors were reverted by a combination of AMG 510 (Figure 3C,D). These findings indicate mutant KRAS decreases the sensitivity of CRC cells to anti‐PD‐1 therapy.

Furthermore, we explored whether mutant KRAS might impair the efficacy of adoptive cell‐transfer (ACT) therapy in CRC patient‐derived xenograft (PDX) models implanted in immunocompromised NOD.SCID mice (Figure 3E). As indicated by tumor volume and weight, ACT therapy in KRAS wild‐type PDXs efficiently inhibited tumor growth (Figure 3F). By contrast, the tumor growth was only slightly lessened by ACT in the PDXs with KRAS^G12C^, while the combination of AMG 510 and ACT resulted in significant antitumor effects (Figure 3G). In light of monitoring by flow cytometry, the distribution of transferred cells was analyzed at 1, 3, 5, 7, 14, and 21 days after transfer. Results at 1, 3, 5, and 7 days after transfer demonstrated similar in vivo accumulation of transferred cells from KRAS wild type versus mutant patients (Figure 3H,I). At later time points, transferred cells from KRAS wild‐type cases had significantly better persistence in the tumors (Figure 3H,I), whereas the cells from KRAS mutant patients declined over time (Figure 3J), and AMG 510 prolonged their persistence in vivo (Figure 3K). These findings were in accord with the differential efficacy of ACT in KRAS mutant versus wild‐type PDX tumors. Thus, we conclude that mutant KRAS has the potential to impair ACT efficacy.

### 2.4 Tumor‐Specific Cytotoxic T‐Cells in KRAS Mutant CRC are Susceptible to Activation‐Induced Cell Death (AICD)

**Figure 4:**
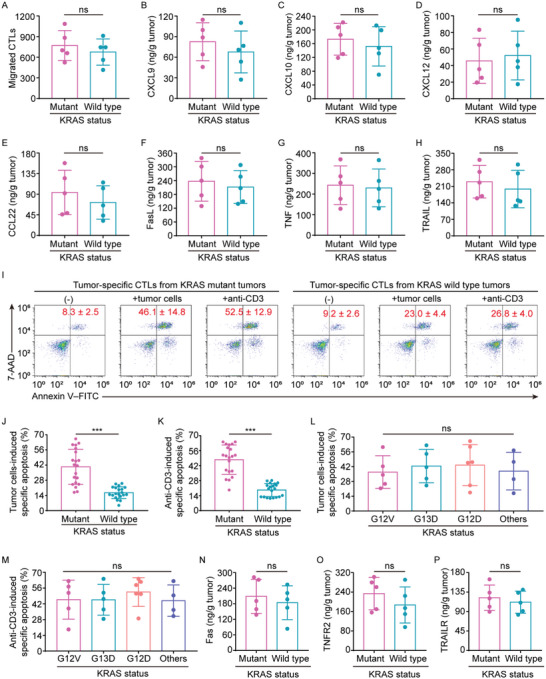
Susceptibility of tumor‐specific CTLs to AICD in KRAS mutant CRC. A) Flow cytometry analysis of the migratory abilities of CTLs from KRAS wild type versus mutant tumors (n = 5). B–E) ELISAs for CXCL9, CXCL10, CXCL12, and CCL22 expression in KRAS wild type versus mutant tumors (n = 5). F–H) ELISAs for FasL, TNF, and TRAIL expression in KRAS wild type versus mutant tumor cells (n = 5). I) Tumor‐specific CTL apoptosis induced by autologous primary tumor cells or anti‐CD3. Numerical values denote the percentage of annexin V^+^ cells (mean ± SD). J,K) Statistics of autologous tumor cells‐ or anti‐CD3‐induced specific apoptotic rates of tumor‐specific CTLs from KRAS wild type versus mutant tumors (n = 20). L,M) Statistics of autologous tumor cells‐ or anti‐CD3‐induced specific apoptotic rates of tumor‐specific CTLs from 20 tumors of different types of KRAS mutations (Others include KRAS G12C (n = 2), G12R (n = 1), and G13C (n = 1) mutations). N–P) ELISAs for Fas, TNFR2, and TRAILER expression in CTLs from KRAS wild type versus mutant tumors (n = 5). ***P ≤ 0.001, and ns indicates *P* > 0.05, by two‐tailed Student's *t*‐test (A–H,J,K,N–P) or one‐way ANOVA (L,M).

Next, we attempted to understand the underlying mechanisms of cytotoxic CD8^+^ T‐cell reduction resulting from mutant KRAS. As shown in Figure 3H,I, adoptively transferred T‐cells from KRAS mutant versus wild‐type patients had similar in vivo accumulation, but poor persistence. In line with this, transwell experiments revealed that tumor‐infiltrating CD8^+^ T‐cells from fresh CRC tissues with mutant KRAS versus those with wild‐type KRAS had similar migrating abilities (**Figure** **4**A). Additionally, KRAS mutant versus wild‐type tumors had comparable expression levels of T‐cell migration‐related chemokines including CXCL9, CXCL10, CXCL12, and CCL22 (Figure 4B–E). These results suggest the mutant KRAS‐driven reduction of tumor‐infiltrating cytotoxic CD8^+^ T‐cells is unlikely to correlate with their recruitment.

Given the role of activation‐induced cell death (AICD),^[^ ^17^, ^18^ ^]^ we enquired whether AICD was involved in the decrease in cytotoxic CD8^+^ T‐cells infiltrating in KRAS mutant tumors. To address this question, tumor cells and tumor‐specific cytotoxic T‐cells (CTLs) were purified from primary tumors with EpCAM^+^ microbeads and anti‐CEA pentamer, respectively. Despite comparable expression of AICD mediators in T‐cells (FasL, TNF, and TRAIL) from KRAS mutant versus wild‐type tumors (Figure 4F–H), markedly increased apoptosis was observed in the tumor‐specific CTLs from KRAS mutant tumor tissues after cells were cocultured with autologous primary tumor cells (Figure 4I,J). In agreement, treatment with anti‐CD3 resulted in massive apoptosis in tumor‐specific CTLs from KRAS mutant, but not wild type, CRC (Figure 4I,K), indicating that tumor‐specific CTLs in KRAS mutant CRC had increased sensitivity to AICD. In addition, similar apoptosis was found in tumor‐specific CTLs from tumors with different types of KRAS mutations (Figure 4L,M), suggesting that their AICD sensitivity was irrespective of KRAS mutation type. These findings were in line with intratumoral cytotoxic CD8^+^ T‐cell reduction in KRAS mutant CRC. However, tumor‐specific CTLs from the KRAS mutant versus wild‐type tumor tissues had matched expression levels of the death receptors, including Fas, TNFR2, and TRAILER (Figure 4N,O), suggesting that the differential AICD sensitivity is not controlled by the death receptor expression, but rather is determined by other factors.

### 2.5 Lactic Acid Sensitizes Activated T‐Cells to AICD via NF‐*κ*B Inactivation

**Figure 5:**
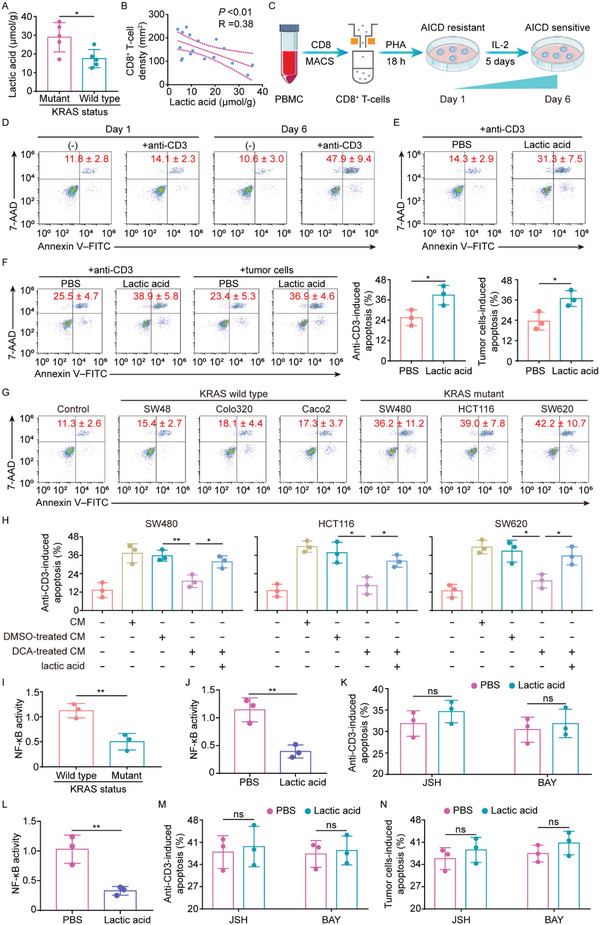
Lactic acid sensitizes activated T‐cells to AICD via NF‐*κ*B inactivation. A) Lactic acid levels in KRAS wild type versus mutant tumors (n = 5). B) Correlation analysis between lactic acid levels and the number of CD8^+^ T‐cells in KRAS mutant CRC tissues (n = 17). C) Scheme of the AICD sensitivity switch during T‐cell activation. D) Representative plots of apoptosis of PHA‐activated CD8^+^ T‐cells with or without anti‐CD3 treatment. Numerical values (mean ± SD) denote the percentage of annexin V^+^ cells (n = 3). E) Day‐6 CD8^+^ T‐cells were pretreated with lactic acid for 12 h, then subjected to anti‐CD3 treatment. The plots represent anti‐CD3‐induced apoptosis (n = 3). F) Tumor‐specific CTLs from KRAS wild‐type tumors were pretreated with lactic acid for 12 h, then subjected to autologous primary tumor cells or anti‐CD3 treatment. The plots represent tumor cells‐ or anti‐CD3‐induced apoptosis (n = 3). G) Anti‐CD3‐induced apoptosis of Day‐6 CD8^+^ T‐cells treated with CM from the indicated cell lines (n = 3). H) SW480, HCT116, and SW620 cells were cultured in the presence or absence of 2 mm DCA for 72 h. CM was collected after the cells were cultured for another 24 h without DCA. In addition, lactic acid levels in CM obtained from SW480, HCT116, and SW620 cells treated with DCA were adjusted to the levels in untreated CM by the addition of lactic acid. Day 6 CD8^+^ T‐cells were treated with the indicated CM and then were analyzed for anti‐CD3‐induced apoptosis (n = 3). I) NF‐*κ*B activity in the CTLs from KRAS wild type versus mutant tumors (n = 3). J) NF‐*κ*B activity in Day‐1 CD8^+^ T‐cells treated with PBS or lactic acid (n = 3). K) Day‐1 CD8^+^ T‐cells were pretreated with JSH or BAY in combination with PBS or lactic acid, then subjected to anti‐CD3 stimulation. The plot represents anti‐CD3‐induced apoptotic rates (n = 3). L) NF‐*κ*B activity in KRAS wild‐type tumors‐derived tumor‐specific CTLs treated with PBS or lactic acid (n = 3). M,N) KRAS wild‐type tumors‐derived tumor‐specific CTLs were pretreated with JSH or BAY in combination with PBS or lactic acid, then subjected to anti‐CD3 or autologous primary tumor cell stimulation. The plot represents anti‐CD3‐ or tumor cells‐induced apoptotic rates (n = 3). *P ≤ 0.05, **P ≤ 0.01, and ns indicates *P* > 0.05, by two‐tailed Student's *t*‐test (A,F,I–L), Person's correlation analysis (B), or one‐way ANOVA (H).

We subsequently explored how such aberrant AICD was triggered in KRAS mutant CRC. We previously revealed that mutant KRAS‐expressing tumor cell‐derived lactic acid played significant roles in the tumor microenvironment engagement and confirmed that mutant KRAS had the ability to directly drive high‐level lactic acid production.^[^ ^12^ ^]^ These findings were further verified herein, which showed significantly higher lactic acid concentrations in KRAS mutant versus wild‐type tissues (**Figure** **5**A; Figure S4A, Supporting Information). Moreover, KRAS mutant CRC exhibited an inverse correlation between lactic acid concentrations and tumor‐infiltrating cytotoxic CD8^+^ T‐cells (Figure 5B). On these grounds, we hypothesized that mutant KRAS‐driven lactic acid might contribute to AICD susceptibility of tumor‐specific CTLs in KRAS mutant CRC. To test this possibility, peripheral blood‐derived T cells were stimulated with PHA for 18 h (Day‐1) and cultured with the cytokine IL‐2 for an additional 5 days (Day‐6) (Figure 5C). Re‐stimulation with anti‐CD3 led to substantial apoptosis in Day‐6 CD8^+^ T‐cells (AICD‐sensitive), but not in Day‐1 CD8^+^ T‐cells (AICD‐resistant) (Figure 5D). In AICD‐resistant Day‐1 T‐cells, treatment with exogenous lactic acid was sufficient to increase AICD (Figure 5E). In parallel, lactic acid could enhance the sensitivity to AICD induced by anti‐CD3 or autologous tumor cells in tumor‐specific CTLs from CRC patients with wild‐type KRAS (Figure 5F). Moreover, conditioned medium (CM) from KRAS mutant, but not wild type, tumor cells increased the sensitivity of Day‐1 T‐cells to AICD (Figure 5G), but this effect was significantly blocked when KRAS mutant tumor cells were pretreated with sodium dichloroacetate (DCA) to abolish lactic acid production (Figure S4B, Supporting Information; Figure 5H). In addition, CM from DCA‐pretreated KRAS mutant tumor cells acquired the ability to sensitize T‐cells to AICD upon the addition of exogenous lactic acid (Figure 5H). Taken together, these findings suggest that lactic acid increases the AICD sensitivity of T‐cells in KRAS mutant CRC.

Next, we aimed to understand how lactic acid affected AICD sensitivity. Notably, NF‐*κ*B signaling determines the sensitivity to T‐cell AICD^[^ ^19^ ^]^ and is shown to be a downstream target of lactic acid.^[^ ^20^ ^]^ Indeed, decreased NF‐*κ*B activation was observed in tumor‐specific cytotoxic CD8^+^ T‐cells from KRAS mutant versus wild‐type CRC (Figure 5I). Therefore, we hypothesized that NF‐*κ*B might be involved in the lactic acid‐elicited susceptibility to AICD. In support of this, NF‐*κ*B inhibition dramatically increased the anti‐CD3‐induced apoptosis (Figure S4C, Supporting Information), and treatment with lactic acid resulted in significant inhibition of NF‐*κ*B activation in the AICD‐resistant Day‐1 CD8^+^ T‐cells expressing wild type KRAS (Figure 5J). Furthermore, lactic acid failed to increase AICD sensitivity when AICD‐resistant Day‐1 T‐cells were pretreated with NF‐*κ*B inhibitors JSH and BAY (Figure 5K). A similar pattern was observed in tumor‐specific CTLs from KRAS wild‐type patients (Figure 5L–N). Collectively, we conclude lactic acid causes the T‐cell AICD by NF‐*κ*B inactivation.

### 2.6 Blockade of Lactic Acid Production Improves Immunotherapeutic Efficacy

**Figure 6:**
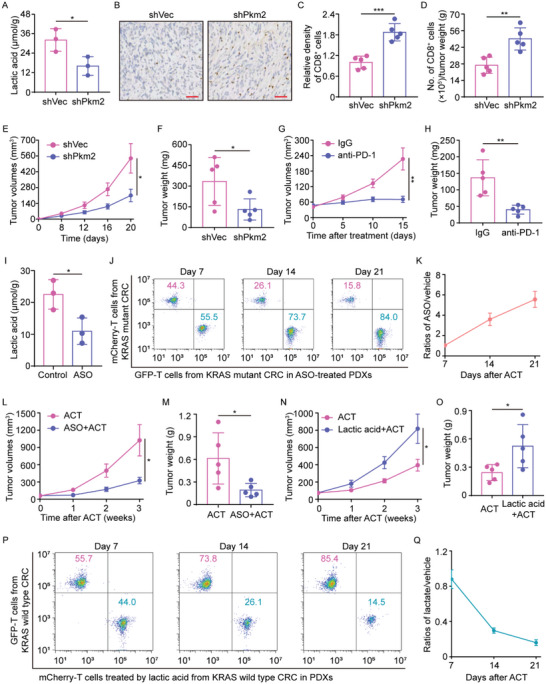
Effects of the lactic acid blockade on immunotherapies. A–F) MC38K cells transfected with shRNA specific for silencing Pkm2 (shVec versus shPkm2) were subcutaneously injected into C57BL/6J mice (n = 5 mice per group). A) Lactic acid levels in shVec‐ versus shPkm2‐tumors. B) Representative IHC staining, C) relative densities, and D) flow cytometry analysis of CD8^+^ cells in shVec‐ versus shPkm2‐tumors. E) Tumor growth curves and F) weights of shVec‐ versus shPkm2‐tumors. G,H) MC38K cells transfected with shPkm2 were subcutaneously injected into C57BL/6J mice. When tumors were palpable, mice were treated with IgG or anti‐PD‐1 (n = 5 mice per group). G) Tumor growth curves and H) weights of tumors with the indicated treatment. I) The lactic acid levels in KRAS mutant PDX tumors treated with Control or ASO specifically targeting PKM2 (n = 5). J,K) GFP‐ or mCherry‐transduced tumor‐reactive T‐cells were transferred into KRAS mutant PDXs treated with or without ASO (n = 3 mice per group). At 7, 14, and 21 days after transfer, KRAS mutant PDXs were mixed with an equal weight of ASO‐treated PDXs, and then transferred cells were recovered from the pooled tissues using a CD8^+^ T Cell Isolation Kit. Flow cytometry was used to determine the proportion of GFP‐ or mCherry‐transduced T‐cells in CD8^+^ cells. L) Tumor growth curves and M) weights of tumors treated with ACT in combination with ASO (n = 5 mice per group). N–Q) Tumor‐reactive T‐cells from CRC patients with wild‐type KRAS were transduced with GFP or mCherry in vitro. Before transfer, mCherry‐transduced T‐cells were pretreated with lactic acid. When the PDXs were palpable, GFP‐ or mCherry‐transduced T‐cells were transferred into autologous PDXs. N) Tumor growth curves and O) weights of tumors with the indicated treatment (n = 5 mice per group). P–Q) At the indicated time points, KRAS wild‐type PDXs treated with GFP‐transduced T‐cells were mixed with an equal weight of PDXs treated with mCherry‐transduced T‐cells, and then transferred cells were retrieved from the pooled tissues. Flow cytometry was used to determine the proportion of GFP‐ or mCherry‐transduced T‐cells in purified total CD8^+^ cells (n = 3 mice per group). Scare bars, 50 µm (B). *P ≤ 0.05, **P ≤ 0.01, ***P ≤ 0.001 and ns indicates *P* > 0.05, by two‐tailed Student's *t*‐test (A,C–I,L–O).

As demonstrated above, we were able to establish the significance of lactic acid in tumor immunological escape in vitro. To confirm these findings in vivo, we generated a stable MC38K tumor cell line in which Pkm2, one of the crucial enzymes involved in glycolysis,^[^ ^21^ ^]^ was knocked down to abolish the production of lactic acid,^[^ ^22^ ^]^ and then subcutaneously injected these cells into C57BL/6J mice. As expected, a significantly lower lactic acid concentration was observed in Pkm2‐knockdown tumors compared to those bearing scrambled constructs (**Figure** **6**A). Importantly, Pkm2‐knockdown tumors had a significantly higher tumor‐infiltrating CD8^+^ T‐cell density (Figure 6B–D), and these tumors were markedly smaller (Figure 6E,F). Although the interpretation of these findings is complicated by the likely cell‐intrinsic role of Pkm2 interference, these results are at least in accord with the model where PKM2‐dependent lactic acid production by cancer cells plays a critical role in the tumor infiltrate of cytotoxic CD8^+^ T‐cells. This inspired us to hypothesize that blockade of lactic acid production could improve immunotherapy response. Support for this hypothesis came from our data in which significant antitumoral activity of anti‐PD‐1 treatment was shown in Pkm2‐knockdown tumors (Figure 6G,H), an effect not seen in MC38K tumors (Figure 3C,D). These findings from MC38K tumors were further confirmed by another model with lactate dehydrogenase (LDH) inhibition by LDH‐IN‐1 to block the production of lactic acid (Figure S5A, Supporting Information). However, the blockade of lactic acid production had limited effects on the tumor infiltrate of cytotoxic CD8^+^ T‐cells, and failed to synergize with immunotherapy in the Kras wild‐type MC38 tumors (Figure S5B,C, Supporting Information). Together, these results pointed to a view that the blockade of lactic acid production selectively improves the response to anti‐PD‐1 therapy in KRAS mutated tumors.

Furthermore, we explored whether the blockade of lactic acid production by tumor cells might prevent AICD in transferred T‐cells and improve ACT efficacy in CRC PDX models. Intratumoral administration of anti‐sense oligonucleotide (ASO) specifically targeting PKM2 in the CRC PDX models with mutant KRAS effectively decreased lactic acid concentrations (Figure 6I). Importantly, treatment with ASO improved the in vivo persistence of transferred cells in KRAS mutant tumors (Figure 6J,K), and thus a combination of ASO and adoptive transfer with tumor‐reactive T‐cells significantly inhibited tumor growth (Figure 6L,M). As a converse correlate to these results, lactic acid pretreatment in transferred cells effectively impaired their anticancer efficacy (Figure 6N,O). More importantly, pretreatment with lactic acid significantly reduced the in vivo persistence of transferred cells in the KRAS wild‐type CRC PDX models (Figure 6P,Q). Collectively, these data suggest lactic acid blockade enables a prolonged antitumor immune response, and points to an encouraging therapeutic avenue for the treatment of patients with KRAS mutant CRC.

## 3 Discussion

The most widely studied mode of action for mutated KRAS is the cell‐intrinsic mechanism involved in human cancer pathogenesis. Recent research indicated that KRAS mutation was associated with several immune cell types in the tumor microenvironment.^[^ ^23^, ^24^, ^25^ ^]^ This work further reinforced this concept by demonstrating a cell‐extrinsic role of mutant KRAS in crosstalk with cytotoxic CD8^+^ T‐cell tumor infiltration in the context of CRC progression. We demonstrated mutant KRAS had the ability to drive a reduction in cytotoxic T‐cell tumor infiltration, and thus mutated KRAS‐expressing tumors had poor response to immunotherapies. Mechanistic investigations showed mutant KRAS‐driven lactic acid increased the susceptibility to AICD of tumor‐specific cytotoxic T‐cells via NF‐*κ*B inactivation, which might underlie the correlation between KRAS status and intratumoral cytotoxic CD8^+^ T‐cells. The rational combination of immunotherapies and the lactic acid blockade was sufficient to establish a long‐term tumor‐specific T‐cell response in KRAS mutant tumors; this is of obvious therapeutic significance.

The cell‐autonomous roles of KRAS in CRC pathogenesis have been clearly demonstrated.^[^ ^7^, ^26^ ^]^ This work further proposed a previously undescribed association between oncogenic KRAS and cancer immunology by clinical sample analysis. These findings were confirmed by subcutaneous models in immune‐competent mice, PDX models in immunocompromised mice, and genetically engineered mouse models, which represent one of the best set‐ups to investigate tumor‐immune interactions. In addition, we found cytotoxic CD8^+^ T‐cell tumor infiltrate was unlikely to correlate with the KRAS mutation type, which suggests that the findings of tumor immune evasion might be a general feature of KRAS mutant CRC. The present findings improve the understanding of the link between oncogenic KRAS and the immune tumor microenvironment.

Microsatellite status is a well‐identified factor related to immune infiltration.^[^ ^27^ ^]^ However, its clinical power remains to be debated. Pagès and colleagues^[^ ^28^ ^]^ revealed that only 45% of patients with MSI tumors and up to 21% of cases with MSS tumors had a high level of immune infiltrate. These insights suggest that the immune infiltrate varied widely across the patients, and might be affected by multiple factors, including tumor genetic features, the genetic background of the patient, and tumor immunoenvironment, each of which is variable across individuals and may influence each other. Our current and previous studies^[^ ^12^ ^]^ identified that KRAS status represents an independent causative factor of tumor‐supporting immunoenvironment that is irrespective of KRAS mutation type, but positively correlates with mutated KRAS protein expression. These results reflect the complexity of the tumor itself and provide a straightforward explanation for a previous report by Taieb and colleagues^[^ ^29^ ^]^ which showed KRAS mutations are independently related to poor prognosis in patients with MSS tumors.

Tumor immunosurveillance is a process where tumor‐specific cytotoxic T‐cells can identify and destroy cancer cells. Paradoxically, activated T‐cells are subjected to immunological elimination by a process termed AICD,^[^ ^18^ ^]^ which can be exploited by tumors to escape immunological destruction. Herein, we observed that tumor‐specific cytotoxic T‐cells in KRAS mutant tumors were more sensitive to tumor‐mediated AICD, and thus displayed poor persistence. The present study provided an explanation for this phenomenon by showing that mutant KRAS‐driven lactic acid contributed to excessive AICD by triggering NF‐*κ*B inactivation. Previous studies ascribed differential AICD sensitivity to cell‐intrinsic abnormalities, including lncRNA NKILA,^[^ ^30^ ^]^ exosomal membrane TNF,^[^ ^31^ ^]^ or hematopoietic progenitor kinase 1.^[^ ^19^ ^]^ The overlap of these findings is that the mechanisms regulating AICD sensitivity converge on cell‐intrinsic NF‐*κ*B activation in T‐cells.

A salient feature of KRAS mutant tumors is the elevated production of lactic acid, which leads to accumulating concentrations of lactic acid in the tumor microenvironment and elevates tumor acidity.^[^ ^12^, ^32^, ^33^ ^]^ As a common metabolite, lactic acid has been shown to promote tumor immune evasion associated with regulatory T cells, tumor‐associated macrophages, and myeloid‐derived suppressor cells.^[^ ^22^, ^34^, ^35^ ^]^ Further evidence linking lactic acid with tumor immunoescape comes from our present data in which it is shown the increased lactic acid levels in KRAS mutant CRC could sensitize tumor‐specific CTLs to AICD. To our knowledge, this is the first study regarding the contribution of the lactic acid‐AICD axis to T‐cell fate decision. The results from our study as well as previous reports^[^ ^34^, ^36^, ^37^ ^]^ pointed out to an immune suppressive role of lactic acid.

Currently, cancer immunotherapies, especially immune checkpoint blockade and ACT, represent a new paradigm in tumor management.^[^ ^38^, ^39^ ^]^ Despite considerable advances, they provide only limited clinical benefits to the majority of CRC patients.^[^ ^40^ ^]^ A major hurdle for satisfactory immunotherapies is that the tumor immunoenvironment lacks sufficient cytotoxic T‐cell tumor infiltration.^[^ ^41^, ^42^, ^43^ ^]^ This study showed that mutant KRAS caused poor persistence of tumor‐infiltrating cytotoxic T‐cells, which was in accord with poor immunotherapy efficacy. Concordant with previous findings,^[^ ^23^ ^]^ we showed that KRAS inhibition could enhance the therapeutic effects of anti‐PD1 treatment. Furthermore, this work demonstrated that lactic acid production by tumor cells represented one of the several mechanisms by which mutant KRAS shapes tumor immunity. Therefore, targeting lactic acid production might serve as a promising strategy for antitumor immunotherapy. The present study reinforced this therapeutic strategy by showing improved ACT upon blockade of lactic acid production. The fact that these findings were done in a humanized model of CRC PDXs increased the clinical translatability of our discovery. As a converse correlate to our findings, a study by Kumagaiin and colleagues demonstrated that lactic acid production resulting from Myc‐mediated LDHA upregulation was associated with anti‐PD‐1 treatment resistance.^[^ ^36^ ^]^ Together, we concluded that KRAS inhibition or blockade of lactic acid production represents an exploitable approach to improve cancer immunotherapies.

One point should be particularly put forward is that KRAS mutant versus wild‐type PDX tumors had a comparable accumulation of transferred cells at day 7 after the transfer, but a significant difference in ACT response at this time point. This disagreement highlights that the accumulation of transferred cells may be not the sole factor that contributes to ACT response difference, and there might exist other concomitant stimulation factors participating in KRAS‐related immunoescape. As described by Wei and colleagues,^[^ ^16^ ^]^ the response of ACT therapy can also be limited by the dysfunction of transferred T cells. Further efforts are required to understand whether dysfunction of transferred T cells contributes to ACT response difference between KRAS mutant and wild‐type tumors.

In conclusion, this work established a link between mutated KRAS and cytotoxic CD8^+^ T‐cell tumor infiltration. This work illuminated the potential clinical utility of interfering with KRAS‐related pathways, possibly representing a promising avenue for improving immunotherapy efficacy.

## 4 Experimental Section

Detailed procedures are provided in Supporting Information.

### Patients and tissue samples

CRC tissues were obtained from patients who had undergone radical surgery at the Sixth Affiliated Hospital of Sun Yat‐sen University (Guangzhou, China). KRAS status was determined by Sanger sequencing. Pathologists determined patients’ status as mismatch‐repair‐deficient (dMMR) or mismatch‐repair‐proficient (pMMR) based on immunohistochemistry staining for four DNA mismatch repair proteins (MLH1, MSH2, MSH6, and PMS2). The procedures for related specimen collections were performed with the approval of the Institutional Review Board of the Sixth Affiliated Hospital of Sun Yat‐sen University (2020ZSLYEC‐238) and informed written consent was obtained from all subjects.

### Immunohistochemistry

Immunohistochemistry (IHC) was done on formalin‐fixed, paraffin‐embedded CRC tissues using anti‐CD8 (Abcam) and anti‐KRAS (MERCK) primary antibodies. CRC tissues were incubated with primary antibodies at 4 °C overnight and then with appropriate secondary antibodies. The slides were stained with 3,3'‐Diaminobenzidine complex and counterstained with hematoxylin. For quantification, the slides were assessed by two independent pathologists who were blinded to the patients’ clinical information. The CD8^+^ cell density was calculated as the number per mm^2^ of CD8^+^ cells on each slide. The KRAS expression levels were determined as the staining intensity (grade 0 for no staining, grade 1 for h weak staining, grade 2 for medium staining and grade 3 for strong staining) multiplied by the grade extents (grade 0 = 0, grade 1 = 1–25%, grade 2 = 26–50%, grade 3 = 51–75% and grade 4>75%).

### Primary‐cell isolation from tumors and peripheral blood

Fresh tumor samples from surgical resection were cut into ≈1 mm^3^ pieces, and then single‐cell suspensions from human tumor tissues were generated. Dead cells were eliminated using a Dead Cell Removal Kit (Miltenyi Biotec). Tumor cells were purified with EpCAM^+^ microbeads (Miltenyi Biotec). Tumor‐specific CTLs within tumors were enriched and purified using anti‐CEA Pentamer‐PE (ProImmune) from HLA‐A2^+^ patients with CRC expressing CEA. Peripheral blood mononuclear cells (PBMCs) were isolated according to the previously reported methods.^[^ ^44^ ^]^ CD8^+^ T‐cells were purified using a human CD8^+^ T‐cell isolation kit (Miltenyi Biotec), and then a complete X‐VIVO‐15 medium (Lonza Walkersville) was used for the ex vivo culture. The cell populations were confirmed to be ≥95% pure by flow cytometric analysis.

### Generation of dendritic cells (DCs) and tumor‐reactive T‐cells from PBMCs

Mononuclear cells were obtained from the peripheral blood of patients with CRC, and cultured for 5 days in VIVO medium containing 100 ng mL^−1^ GM‐CSF and 30 ng mL^−1^ IL‐4. Half of the medium (by volume) was replaced with fresh medium and cytokines every two days. On day 6, DCs were matured through incubation with 10 ng mL^−1^ TNF‐*α* for 24 h and then pulsed for 24 h with autologous primary tumor‐cell lysates by freeze‐thawing with liquid nitrogen (200 µg protein/1 × 10^6^ cells mL^−1^) to generate autologous tumor antigen‐loaded DCs. To obtain tumor‐reactive T‐cells, CD8^+^ T‐cells were isolated from the peripheral blood of the same donors using a CD8^+^ T Cell Isolation Kit (Miltenyi Biotec), and then co‐cultured with autologous tumor antigen‐loaded DCs at a ratio of 5:1 in VIVO medium supplemented with 25 IU mL^−1^ IL‐2 for 5 days.

### Transduction of T‐cells

Lentiviral vectors were used for transduction with mCherry or GFP plasmid into T‐cells. To generate lentiviruses, 293T cells were transfected with lentiviral and package vectors by PEI. Virus‐containing supernatants were collected 48 and 72 h after transfection and concentrated by centrifugation at 1500 × g in ultrafiltration tubes. CD8^+^ T‐cells were stimulated with plate‐bound 2 µg mL^−1^ anti‐CD3/CD28 antibodies in the presence of 100 IU mL^−1^ IL‐2 for 24 h. Activated T‐cells were cultured with concentrated lentivirus (multiplicity of infection of 25) supplemented with 8 µg mL^−1^ polybrene, centrifuged at 850 × g for 80 min at 32 °C, and cultured for 9 h. The infection procedure was repeated the next day, and cells were cultured in fresh VIVO medium supplemented with 100 IU mL^−1^ IL‐2.

### Ex vivo induction and evaluation of activation‐induced cell death

Freshly isolated CD8^+^ T‐cells from PBMCs were activated by phytohaemagglutinin (PHA, 1 µg mL^−1^; Sigma) for 18 h and then cultured with the cytokine IL‐2 (25 IU mL^−1^; PeproTech) for an additional 5 days. To induce activation‐induced cell death (AICD), PHA‐activated CD8^+^ T‐cells were treated with anti‐CD3 (10 µg mL^−1^; BD Bioscience), and tumor‐specific CTLs were stimulated with anti‐CD3 (10 µg mL^−1^; BD Bioscience) or autologous tumor cells pre‐dyed by CellTracker Deep Red Dye (Thermo Fisher Scientific) at a 1:1 ratio for 18 h at 37 °C. Anti‐CD3‐induced AICD was determined by flow cytometry analysis using annexin V/7‐AAD staining kits (MULTISCIENCES). For tumor cell‐induced AICD, cocultures of tumor‐specific CTLs and autologous tumor cells were subjected to flow cytometric cell sorting to exclude tumor cells and retrieve tumor‐specific CTLs and then purified CTLs were stained with annexin V/7‐AAD, followed by flow cytometry analysis. To investigate the effects of specific signaling pathways on AICD, cells were pretreated with Control (DMSO), 6 mM JSH‐23 (MedChemExpress), or 2 mm Bay11‐7082 (MedChemExpress) for 1 h, or 10 mm lactic acid (Sigma) for 12 h, or conditioned medium (CM) from the indicated cells for 24 h at 37 °C. Apoptotic cell death was tested by flow cytometry using an apoptosis detection kit (Multiscience) that stains for annexin V and 7‐AAD. The apoptotic cell percentages included the percentages of early (annexin V^+^7‐AAD^–^) and late apoptotic cells (annexin V^+^7‐AAD^+^). Specific apoptosis was described in the previous report.^[^ ^31^ ^]^

### Flow cytometry analysis

For surface marker analysis, single‐cell suspensions from the indicated samples were labeled with the indicated fluorescein‐conjugated antibodies at 4 °C for 25 min. For apoptosis analysis, cells were collected by centrifugation, incubated with 5 µL Annexin V in 100 µL binding buffer for 10 min at room temperature, dyed with 7‐AAD, and then immediately analyzed by flow cytometry. The fluorescence signals for the indicated samples were detected using a Beckman CytoFLEX. FlowJo10 software was used to analyze the data.

### In vivo mouse studies

This study included the following mouse strains: C57BL/6J, NOD‐SCID, Apc^flox/+^, LSL‐Kras^G12D/+^, and Villin‐Cre^ERT2^. Animals were maintained in pathogen‐free conditions at the Experimental Animal Center of Sun Yat‐sen University. All animal work was conducted under the protocols approved by the Institutional Animal Care and Use Committee of Sun Yat‐sen University (SYSU‐IACUC‐2020‐000370 and SYSU‐IACUC‐2021‐000285).

To generate the genetically engineered CRC mouse model, Apc^flox/+^ mice were crossed with LSL‐Kras^G12D^ and the tamoxifen‐inducible Villin‐Cre^ERT2^ transgenic mice to obtain mice with Villin‐Cre^ERT2^Apc^flox/+^ or Villin‐Cre^ERT2^Kras^G12D/+^Apc^flox/+^. Genotyping was performed by tail DNA PCR using specific primers. When mice were at the age of 8 weeks, 1 mg mL^−1^ 4‐hydroxytamoxifen (4‐OHT) was introduced into the adult colon via enema. All mice were sacrificed 10 weeks later, and the colonic tumors were subjected to gross inspection, IHC analysis, or flow cytometry.

In C57BL/6J mice (6 weeks old), 6 × 10^5^ MC38 cells with stable transfection of empty vector or cDNA encoding the KRAS^G12C^ mutation were subcutaneously injected at day 0. In NOD‐SCID mice (6 weeks old), CRC tissues were harvested from patients with KRAS^G12C^ mutation, and the PDX model was established according to the previous description.^[^ ^45^ ^]^ When the tumors were palpable, tumor‐bearing mice were randomly assigned to the indicated treatments. The KRAS^G12C^ inhibitor AMG 510 (Selleck) was given daily through oral gavage at 30 mL kg^−1^. Anti‐PD‐1 monoclonal antibody (Bioxcell) and IgG isotype control (Bioxcell) were administered once every 3 days via intraperitoneal injections at a dosage of 200 mg per injection. Tumor growth was monitored by digital calipers, and the tumor volumes were recorded using the following formula: Volume = (longer diameter × shorter diameter^2^)/2.

### Adoptive cell‐transfer therapy

In ACT therapy for PDX tumors, tumor‐reactive T cells and autologous tumor antigen‐loaded DCs were first prepared as indicated above. To track the distribution of transferred cells, tumor‐reactive T cells were transduced with GFP‐ or mCherry‐tagged vector before transfer. After transfection, 2.5 × 10^6^ tumor‐reactive T‐cells and 0.5 × 10^6^ DCs were intravenously transfused into each PDX‐bearing mouse via the tail vein after palpable tumor formation. At the indicated time points, PDXs were harvested to determine the presence of transferred cells in vivo by flow cytometry. Tumor growth was monitored and recorded as described above.

### Statistics analysis

Statistical comparisons were performed by GraphPad Prism Software. Data were presented as mean ± standard deviation (SD) except were stated otherwise. Detailed data processing, sample size, and statistical methods for each result were shown in the corresponding figure legends. All *p* values were two‐sided and *p*‐value ≤ 0.05 (ns = not significant, *p ≤ 0.05, **p < 0.01, ***p < 0.001) was considered as statistically significant.

## Conflict of Interest

The authors declare no conflict of interest.

## Author Contributions

Z.W.Z. and L.K. contributed equally to this work as co‐corresponding authors. H.S.L., Z.X.L., and S.J.C. were co‐first authors. H.S.L., Z.X.L., S.J.C., Z.W.Z., and L.K. conceptualized the study. H.S.L., Z.X.L., and S.J.C. developed the experimental methods. L.H., W.X.L., C.Z., X.B.Z., and S.J.L. performed the investigations and data analyses. H.S.L., Z.X.L., and S.J.C. wrote the original draft of the manuscript. Z.W.Z. and L.K. reviewed and edited the manuscript. H.S.L. and L.K. acquired funding and supervised the study.

## Translational Relevance

The cell‐intrinsic mechanisms underlying the role of mutant KRAS in human cancer pathogenesis have been widely studied. Here, we provide key perspectives on the cell‐extrinsic role of oncogenic KRAS. We found KRAS mutation in colorectal cancer drove immune evasion by sensitizing cytotoxic T‐cells to activation‐induced cell death via lactic acid‐mediated NF‐*κ*B inactivation, and impaired the efficacy of anti‐PD‐1 and adoptive T‐cell therapy. Importantly, KRAS inhibition and lactic acid production blockade prolonged transferred T‐cell persistence in vivo, thereby improving the immunotherapeutic efficacy. Our findings identify a potential clinically available strategy for combating mutant KRAS tumors.

## Supporting information

Supporting Information

## Data Availability

The data that support the findings of this study are available from the corresponding author upon reasonable request.
