# Supplementary material for: Mutant KRAS Drives Immune Evasion by Sensitizing Cytotoxic T‐Cells to Activation‐Induced Cell Death in Colorectal Cancer
Source: Adv Sci (Weinh). 2023 Jan 4;10(6):2203757. doi: 10.1002/advs.202203757 (PMC9951350; doi:10.1002/advs.202203757)
Supplement: Supplementary file 1 — Supporting Information [file ADVS-10-2203757-s001.pdf]

# Supporting Information for

# Mutant KRAS drives immune evasion by sensitizing cytotoxic T-cells to activation-induced cell death in colorectal cancer

**Author**: Huashan Liu, Zhenxing Liang, Sijing Cheng, Liang Huang, Wenxin Li, Chi Zhou, Xiaobin Zheng, Shujuan Li, Ziwei Zeng, Liang Kang

**Correspondence**:

Liang Kang, MD, PhD

Email: kangl@mail.sysu.edu.cn

Ziwei Zeng, MD, PhD

Email: zengzw@mail2.sysu.edu.cn

**This Supporting Information Includes:**

Figure S1-5

Supplementary Methods

**Figure S1**

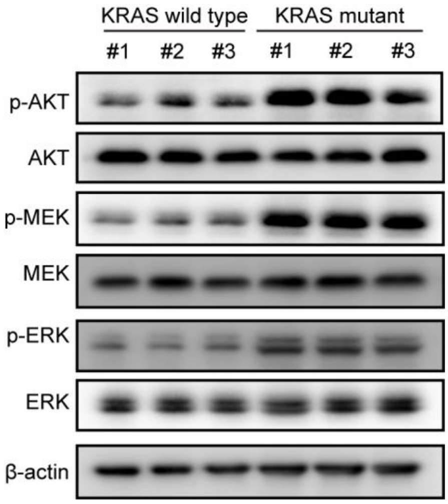


**Figure S1.** Western blots showing RAS signaling in KRAS mutant versus wild type CRC patient samples (n=3 per group).

## Figure S2

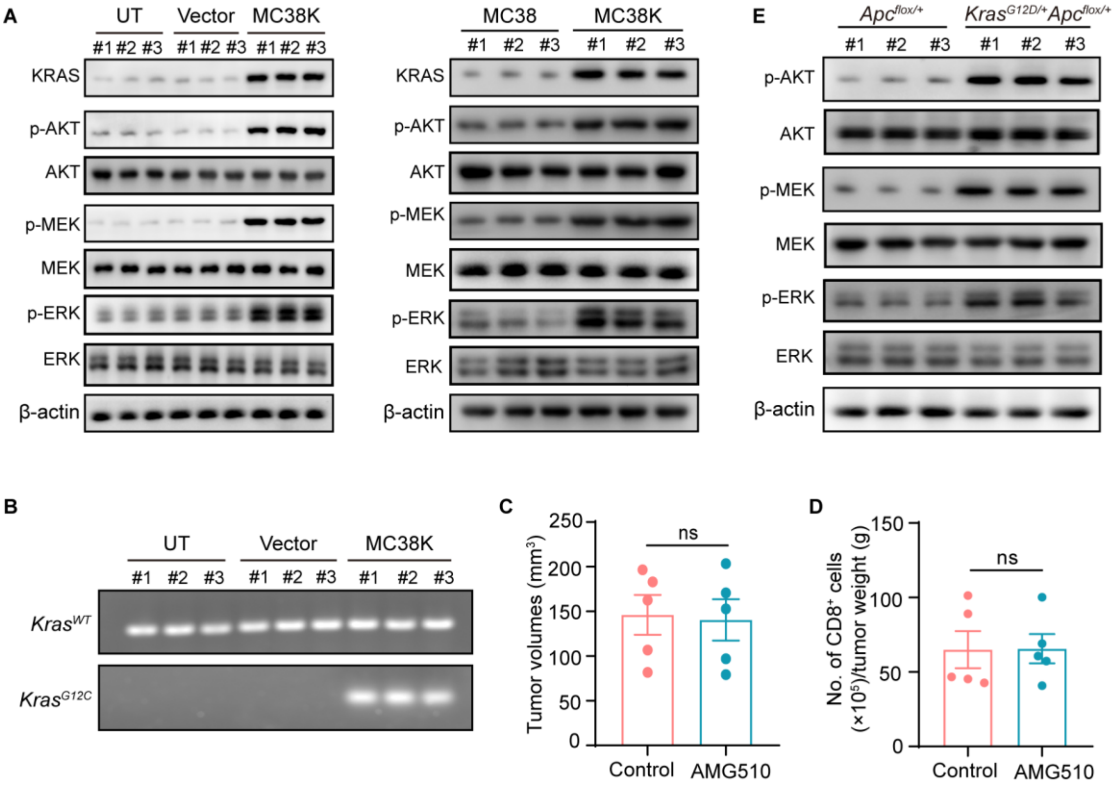


**Figure S2.** (**A**) Western blots showing effects of Kras$^{G12C}$ transfection on Kras expression and Ras signaling in vitro (left) and at the end of subcutaneous xenograft experiments (right). (**B**) RT-PCR gels showing the transfected Kras$^{G12C}$ transcript and endogenous wild type Kras in MC38 versus MC38K cells. (**C-D**) MC38 cells were subcutaneously injected into C57BL/6J mice. When tumors were palpable, mice were treated with vehicle or 30 mg/kg AMG 510 (n=5 mice per group). (**C**) Tumor size and (**D**) flow cytometry analysis of CD8$^+$ cells in tumors measured after 15 days of AMG 510 treatment. (**E**) Western blots showing effects of transgenic Kras$^{G12C}$ on Ras signaling in the intestinal tumors from Villin-Cre$^{ERT2}$Apc$^{flox/+}$ versus Villin-Cre$^{ERT2}$Kras$^{G12D/+}$Apc$^{flox/+}$ mice at week 18 (n=3 mice per group). UT, MC38 cells without any treatment; Vector, MC38 cells transfected with empty vectors; MC38K, MC38 cells with transfection of Kras$^{G12C}$. ns indicates $P > 0.05$, by two-tailed Student's t-test (**C-D**).

**Figure S3**

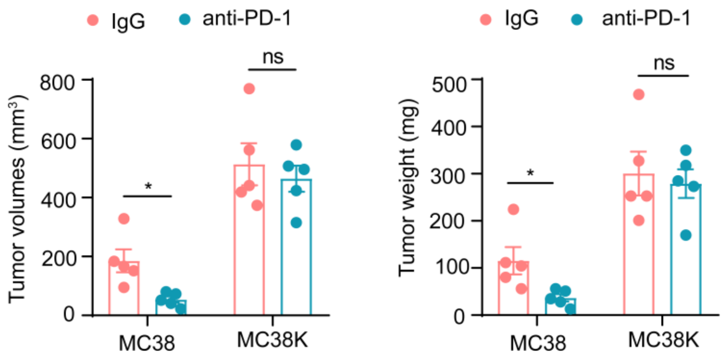


**Figure S3.** MC38 or MC38K cells were subcutaneously injected into C57BL/6J mice. After palpable tumor formation, mice were treated with IgG or anti-PD-1 (n=5 mice per group). Tumor size (left) and weights (right) were measured after 15 days of treatment. *$P \leq 0.05$, and ns indicates $P > 0.05$, by two-tailed Student's t-test.

**Figure S4**

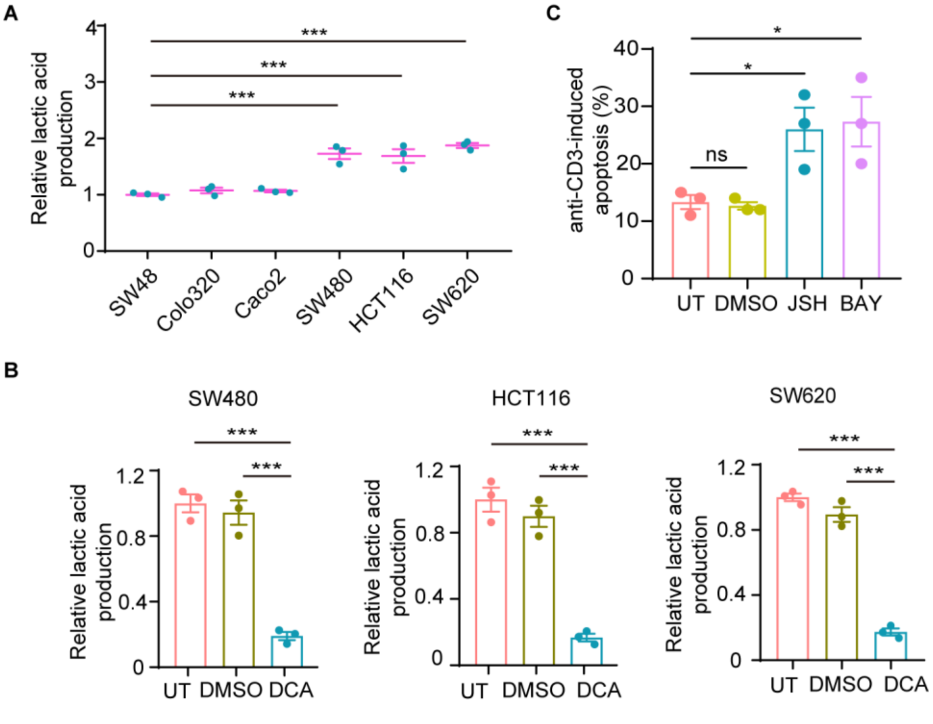


**Figure S4.** (**A**) Relative lactic acid levels in the media of the indicated cells (n = 3). (**B**) SW480, HCT116 and SW620 cells were cultured in the presence or absence of 2mM sodium dichloroacetate (DCA) for 72 h. Afterwards, CM was collected and the lactic acid levels were tested (n=3). (**C**) AICD-resistant Day-1 CD8$^+$ T-cells were pretreated with JSH or BAY, then subjected to anti-CD3 stimulation. The plot represents anti-CD3-induced apoptotic rates (n = 4). UT, untreated; DMSO, vehicle control. *$P \leq 0.05$, **$P \leq 0.01$, ***$P \leq 0.001$ and ns indicates $P > 0.05$, by one-way ANOVA (A-C).

**Figure S5**

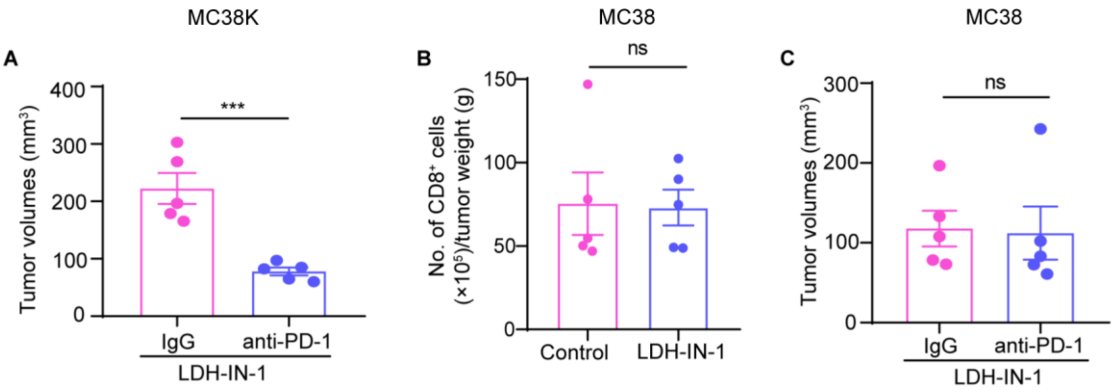


**Figure S5.** (**A**) MC38K cells were subcutaneously injected into C57BL/6J mice. When tumors were palpable, LDH-IN-1 combined with IgG or anti-PD-1 was administrated to MC38K tumor-bearing mice (n=5 mice per group). Tumor size was measured after 15 days of treatment. (**B-C**) MC38 cells were subcutaneously injected into C57BL/6J mice. When tumors were palpable, mice were treated with vehicle or LDH-IN-1 (n=5 mice per group). (**B**) Tumor infiltrate of cytotoxic $CD8^+$ T-cells and (**C**) tumor size was measured after 15 days of treatment. ***$P \leq 0.01$, and ns indicates $P > 0.05$, by two-tailed Student's t-test (A-C).

## Supplementary Methods

### Generation of MC38K cells

The wild-type Kras-expressing MC38 cells were used to generate cells expressing $Kras^{G12C}$ (designated as MC38K). To generate the $Kras^{G12C}$ overexpression construct, the coding sequence of $Kras^{G12C}$ was amplified using ClonExpress II One Step Cloning Kit (Vazyme), and then the cDNA was inserted into the lentiviral expression vector pCDH-CMV-Puro. As controls, the empty vector pCDH-CMV-Puro were used. The constructs were verified by DNA sequencing. For stable transfection, HEK 293T cells were co-transfected with the indicated target plasmids, psPAX2 and pMD2.G using Lipofectamine® 3000 (Invitrogen) to produce lentivirus. Afterwards, MC38 cells were infected with the lentivirus in the presence of polybrene and selected with puromycin. The successful generation of MC38K cells was confirmed by Sanger sequencing, RT-PCR and Western blots.

### Enzyme-linked immunosorbent assay (ELISA)

The indicated tissues or cells were collected for detection of CXCL9, CXCL10, CXCL12, CCL22, FasL, TNF, TRAIL, Fas, TNFR2, and TRAILR. All the detection was performed by ELISA kits from Abcam. All procedures were performed according to the manufacturer's recommendations. The standard curves were generated using ELISACalc software.

### Determination of NF-κB activity

NF-κB activity was measured using a NF-κB p65 Transcription Factor Assay Kit (Abcam) as per the manufacturer's instructions. NF-κB p65 contained in a nuclear extract, binds to the NF-κB p65 response element, and is detected using an anti-NF-κB p65 antibody. A secondary antibody conjugated to HRP is added to provide a colorimetric readout at 450 nm using a Microplate Reader. Each sample was determined in triplicate.

## Determination of lactate concentration

Lactate concentration was measured using a Lactate Assay Kit (BioVision) according to the manufacturer's instructions. Conditioned media were harvested and then diluted 1:400 with lactate assay buffer and prepared as quadruplicates for the colorimetric lactate assay. The absorbance was measured at 570 nm using a Microplate Reader immediately after sample preparation. Background absorbance was subtracted. The intratumoral lactic acid concentration was tested for the tumors after homogenization and methanol extraction, using the colorimetric assay described above.

## Short hairpin RNA (shRNA) construction and transfection

To knock down Pkm2, the shRNA construct (5′-GCCTCCTTCAAGTGCTGCA-3′) was cloned into the pLKO.1 vectors. The pLKO.1 containing a scrambled shRNA was used as the control. HEK 293T cells were transfected with pMD2G, psPAX2 (Addgene) and the indicated target plasmids according to the manufacturer's protocol. The lentivirus medium from HEK 293T cells was added to the indicated cells. After infected with lentivirus, all cell lines were selected with puromycin.
